# Extensive reduction in choroidal thickness after photodynamic therapy in eyes with central serous chorioretinopathy

**DOI:** 10.1038/s41598-023-37802-w

**Published:** 2023-07-05

**Authors:** Naomi Nishigori, Yuki Muraoka, Masaharu Ishikura, Takahiro Kogo, Naoko Ueda-Arakawa, Manabu Miyata, Hiroshi Tamura, Masayuki Hata, Ayako Takahashi, Masahiro Miyake, Akitaka Tsujikawa

**Affiliations:** Department of Ophthalmology and Visual Sciences, Kyoto University Graduate School of Medicine, Sakyo-ku, Kyoto, 606-8507 Japan

**Keywords:** Macular degeneration, Retinal diseases

## Abstract

We examined the effect of reduced fluence (rf)-photodynamic therapy (PDT) of the macular area on the wide-field choroidal thickness in 20 eyes with central serous chorioretinopathy (CSC) and 20 age- and sex-matched control eyes. The choroidal thickness at the posterior pole was measured before and after rf-PDT, using a grid with inner and outer rings, each divided into superotemporal, inferotemporal, superonasal, and inferonasal quadrants, respectively, making up a total of nine subfields including the central 3 mm ring. Before treatment, all eyes showed wide-field choroidal thickening from the dilated vortex vein ampulla to the fovea, along the course of the vein. After rf-PDT of the macular area, the choroidal thickness significantly decreased, not only in the irradiated macular area but also outside the arcade vessels in all quadrants (*p* < 0.001 for all inner subfields; *p* = 0.035 and *p* = 0.024 for the outer superonasal and inferonasal subfields, respectively; *p* < 0.001 and *p* = 0.004 for the outer superotemporal and inferotemporal subfields, respectively). For control eyes, the choroidal thickness did not differ between the initial visit and follow-up 1.2 ± 0.7 months after the initial visit (*p* > 0.05 for all subfields). These findings provide new insights into the pathogenesis of CSC and explain the reasons for the effectiveness of rf-PDT for this condition.

## Introduction

Central serous chorioretinopathy (CSC), predominantly observed in middle-aged men^1^, is characterized by subretinal fluid (SRF) at the posterior pole^2^. Indocyanine green angiography (ICGA) has proven to be useful in CSC to demonstrate choroidal vascular abnormalities such as choroidal filling delay, dilated vasculature^3^, choroidal hyperpermeability, and punctate hyperfluorescent spots^4^. Moreover, optical coherence tomography (OCT)-assisted enhanced-depth imaging (EDI)^5,6^ or swept-source (SS) OCT^7^ has shown macular choroidal thickening in CSC. These observations suggest that the underlying pathogenesis of CSC is primarily related to functional abnormalities of the choroidal vasculature. Macular choroidal thickening associated with choroidal vascular hyperpermeability may be the main cause for the occurrence of SRF at the posterior pole^7^.

The submacular region of the choroid, being a convergence point for several arterial and venous watershed zones, is uniquely susceptible to chronic ischemia compared to the rest of the choroid. This vulnerability is especially significant given that many macular disorders, including macular degeneration, primarily affect this area^8^. The macular region is densely populated with cone photoreceptors that regulate visual activity, necessitating a robust submacular choroidal blood supply^9^. This underscores the critical importance of focusing on the submacular area within the context of the broader choroid.

Recently, wide-field (WF) imaging has demonstrated novel findings associated with CSC, such as vertically and asymmetrically dilated vortex veins^10^ and their anastomosis at the watershed^11^. These findings suggest that abnormal choroidal vascular features in eyes with CSC are not confined to the macula but extend extensively to the periphery. A recent analysis using WF SS-OCT showed that dilated vortex veins were associated with choroidal thickening from the vortex vein ampulla along the course of the vein in eyes with CSC^12^. Moreover, a study using anterior-segment OCT showed increased scleral thickness in eyes with CSC^13–15^. These findings have improved the understanding of the pathogenesis of CSC illustrating that the impaired drainage of the affected vortex veins at the scleral outlet contributes to the choroidal overload, resulting in the formation of SRF at the posterior pole^16^.

Most of the SRF from CSC is spontaneously absorbed. However, persistent SRF may cause poor visual prognosis^17,18^. Currently, photodynamic therapy (PDT) is the treatment of choice for chronic or recurrent CSC^19^. Studies have shown that half-dose (hd) and reduced-fluence (rf)-PDT are both effective and safe treatments for CSC^20–23^. A key benefit of rf-PDT is its ability to control the disease while minimizing risks. Some treatments can harm the choriocapillaris, a layer of tiny blood vessels in the eye, which may cause the retina to become thinner and potentially lead to vision loss. This risk is especially concerning for CSC patients, who generally have good visual acuity that they want to preserve. As such, rf-PDT, which mitigates these risks, is a highly favored treatment choice for this group of patients.

Several studies have reported changes in the CSC-associated choroidal structures after PDT^24–26^. Maruko et al.^26^ showed reduced subfoveal choroidal thickness and choroidal vascular hyperpermeability in CSC after PDT. Presently, PDT is considered to reduce the recurrence of SRF in CSC more effectively than does focal photocoagulation^27^.

However, the effect of PDT, when applied to the macular area on the extensive choroid has not been fully elucidated. Most recently, a study using WF SS-OCT showed that the choroidal thickening extended to the periphery, including vortex vein ampulla^12^. The present study examined the effect of rf-PDT on the WF choroidal thickness in CSC. The current findings may be helpful to elucidate the mechanism of rf-PDT for CSC and improve our understanding of the pathophysiology of CSC.

## Results

**Figure 1:**
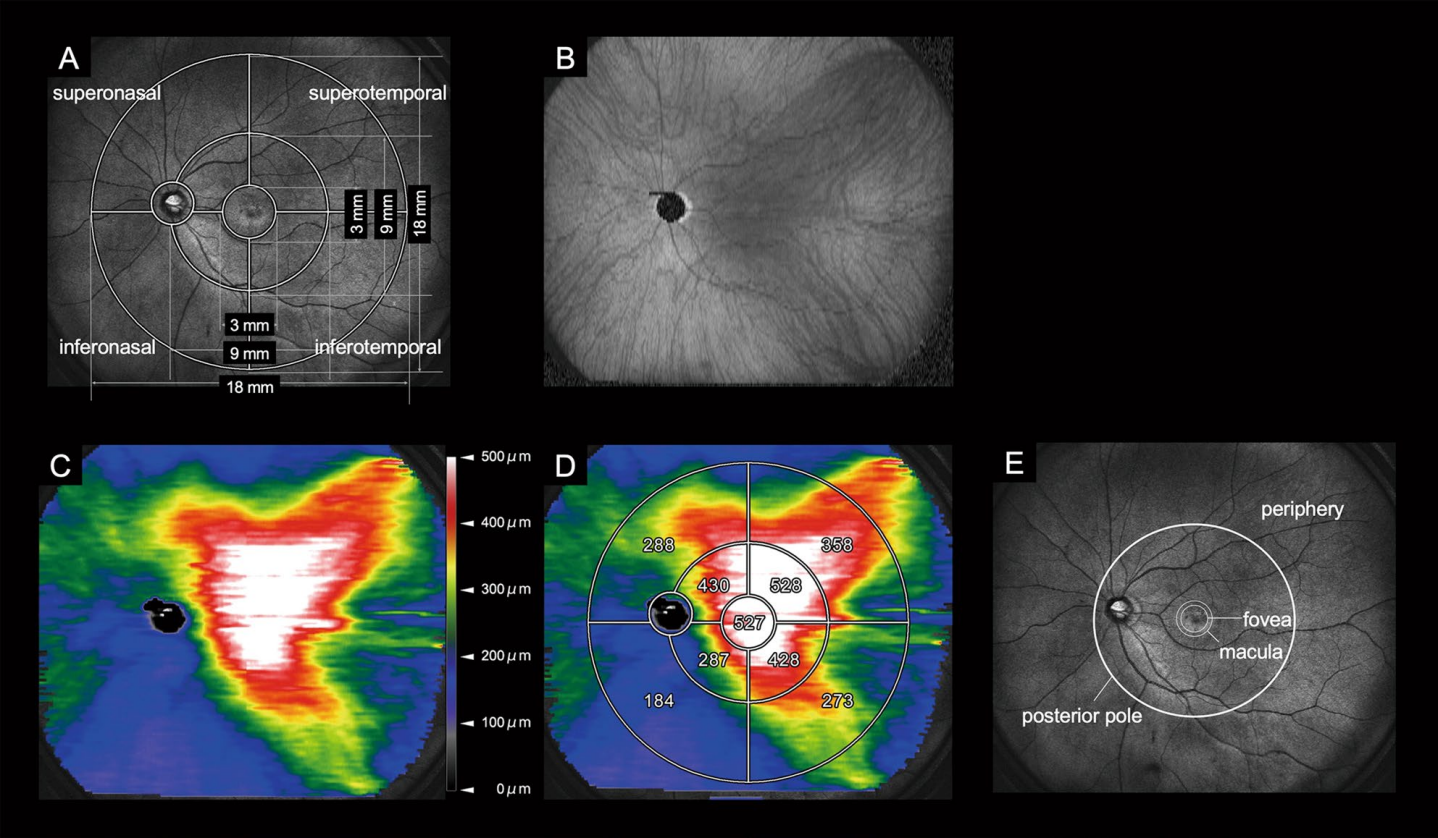
Choroidal thickness map analysis using enhanced depth imaging in wide-field swept-source optical coherence tomography (**A**) Infrared scanning laser ophthalmoscopy images with a measurement grid that was developed for assessing changes in the wide-field (WF) choroidal thickness. The grid consisted of nine subfields divided by three circles with diameters of 3, 9, and 18 mm, respectively, and four lines. The circumferential and zonal areas enclosed by these circles were divided into superotemporal, inferotemporal, superonasal, and inferonasal subfields, considering the arrangement of the vortex veins. (**B**): En face swept-source optical coherence tomography image of the choroid with dilated vortex veins. (**C**): WF choroidal thickness map. (**D**): WF choroidal thickness map with the overlaid grid. The value for each subfield indicates the mean choroidal thickness (mm) in the subfield. (**E**): The foveal, macular, posterior pole, and peripheral areas are illustrated.

This study included 20 eyes of 20 patients (14 men, 6 women) with unilateral CSC treated with rf-PDT, as well as 20 eyes of 20 patients (12 men, 8 women) with unilateral CSC that was untreated for a duration of 1.2 ± 0.7 months (Fig. 1).

**Figure 2:**
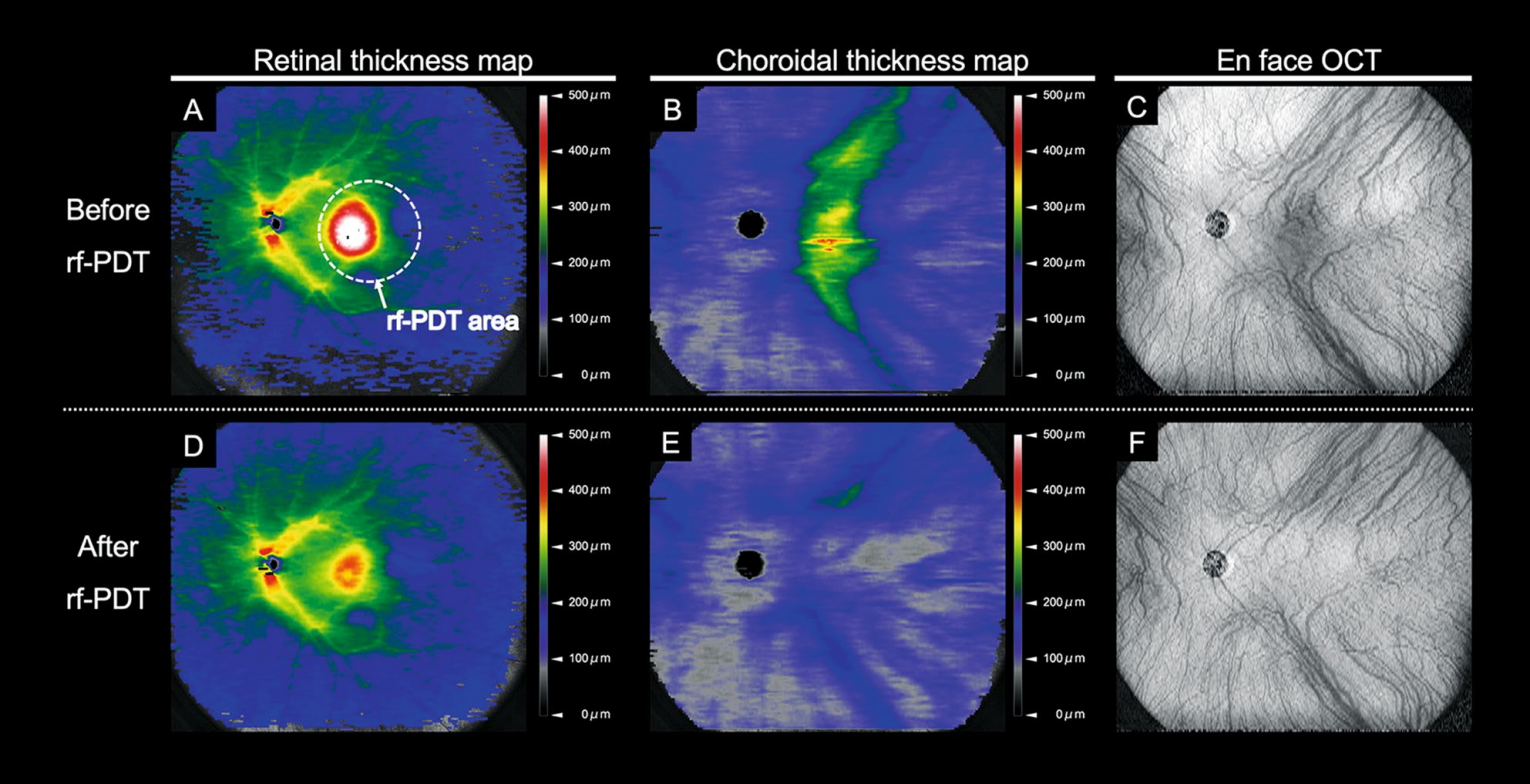
Wide-field retinal and choroidal thickness maps before and after reduced-fluence photodynamic therapy in a representative eye with central serous chorioretinopathy (**A**): Widefield (WF) retinal thickness map before reduced-fluence photodynamic therapy (rf-PDT). At baseline, the Snellen visual acuity (VA) was 20/100. The white dotted circle represents the rf-PDT spot. (**B**): WF choroidal thickness map before rf-PDT. The choroidal thickening extended from the vicinity of the ampulla of the temporal vortex veins to the macula, along the course of the vortex veins. (**C**): En face swept-source optical coherence tomography (OCT) image of the choroid with dilated vortex veins, obtained before rf-PDT. (**D**): WF retinal thickness map, obtained 2 weeks after rf-PDT, showing a decrease in macular thickening. The Snellen VA was maintained at 20/100. (**E**): The WF choroidal thickness map, obtained 2 weeks after rf-PDT, showing that choroidal thickening was resolved not only in the macular area but also outside the vascular arcade. (**F**): En face swept-source OCT image of the choroid obtained 2 weeks after rf-PDT.

**Figure 3:**
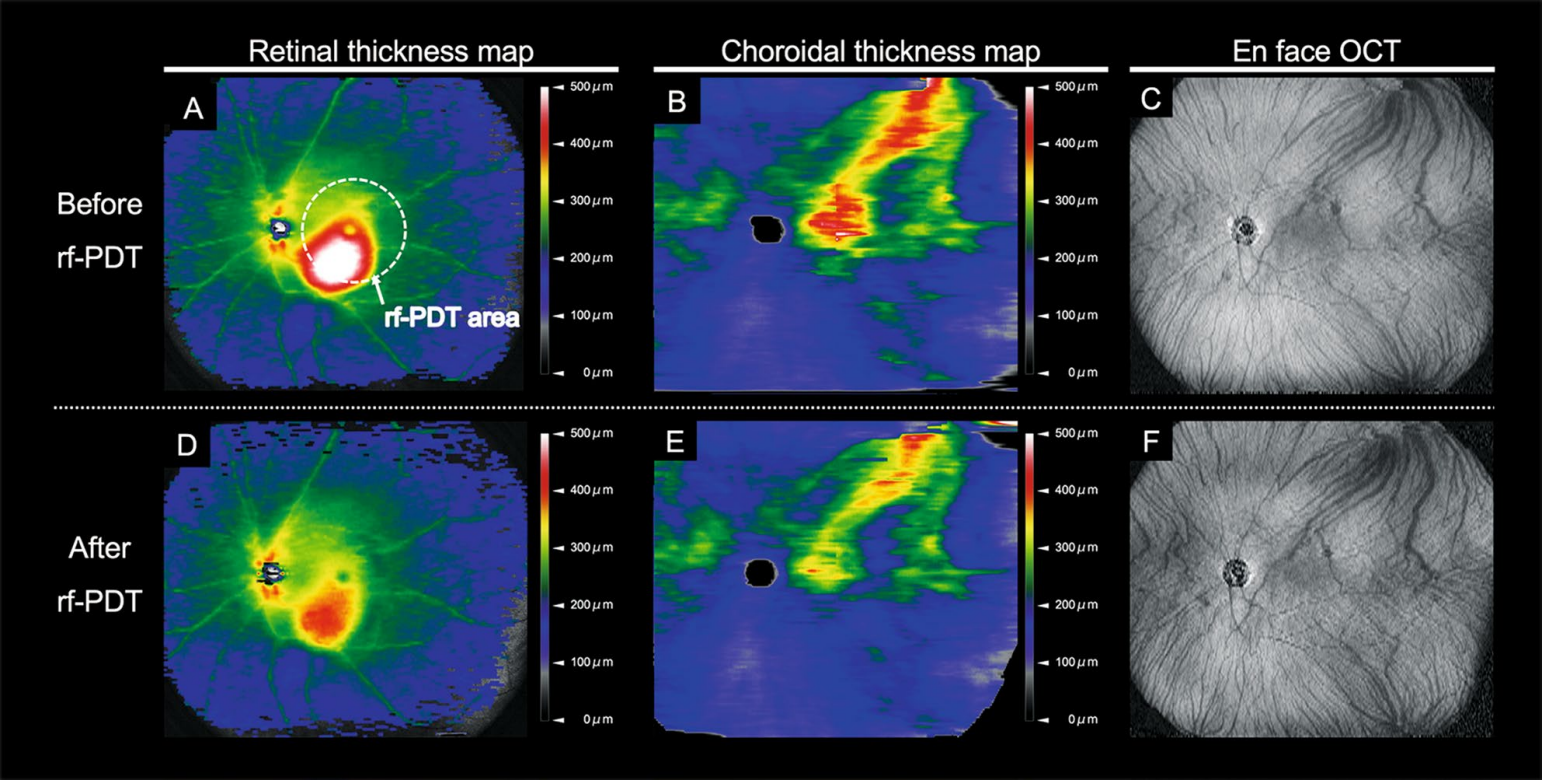
Wide-field retinal and choroidal thickness maps before and after reduced-fluence photodynamic therapy in another eye with central serous chorioretinopathy (**A**): Widefield (WF) retinal thickness map obtained before reduced-fluence photodynamic therapy (rf-PDT). At baseline, the Snellen visual acuity (VA) was 20/25. The white dotted circle represents the rf-PDT spot. (**B**): WF choroidal thickness map obtained before rf-PDT. Choroidal thickening extended from the vicinity of the temporal vortex vein ampulla to the macula along the course of the vortex veins. (**C**): En face swept-source optical coherence tomography (OCT) image of the choroid showing dilated vortex veins before rf-PDT. (**D**): WF retinal thickness map, obtained 3 weeks after rf-PDT, showing a decrease in macular thickening. The Snellen VA improved to 20/20. (**E**): WF choroidal thickness map, obtained 3 weeks after rf-PDT, showing that choroidal thickening was resolved not only in the macular area but also outside the vascular arcade. (**F**): En face swept-source OCT image of the choroid, obtained 3 weeks after rf-PDT.

The choroidal thickness map, captured using WF SS-OCT, covered the vortex vein ampulla. All 40 eyes exhibited some signs of dilated vortex veins and thickening of the choroid from the vortex vein ampulla to the fovea along the course of the vein at the initial visit (Figs. 2 and 3).

Table 1 delineates the baseline characteristics of patients experiencing visual disturbances due to SRF. The mean pre-rf-PDT SRF height at the fovea and the subfoveal choroidal thickness were 151 ± 101 µm and 352 ± 101 µm, respectively, in the rf-PDT group and 140 ± 78 µm and 372 ± 100 µm, respectively, in the control group. There were no significant between-group differences at the initial visit (*p* = 0.70 for foveal SRF height and *p* = 0.25 for subfoveal choroidal thickness; Table 1).

**Table 1:** Baseline characteristics of patients with central serous chorioretinopathy in the control group and the reduced-fluence therapy group.

	Control group (n = 20)	rf-PDT group (n = 20)	*P* value
Number, men/women	12/8	14/6	0.51
Age, years (range)	58.1 ± 15.0 (33–80)	62.5 ± 11.9 (41–80)	0.41
Systolic blood pressure, mmHg	130 ± 22	137 ± 22	0.28
Diastolic blood pressure, mmHg	78 ± 15	86 ± 15	0.10
Symptom duration in the current episode, months	4.5 ± 4.5	14.7 ± 8.5	< 0.001
Spherical equivalent, diopter	− 0.43 ± 0.99	− 0.43 ± 1.25	0.94
Axial length, mm	23.7 ± 1.0	23.6 ± 0.5	0.71
Visual acuity in logMAR	0.20 ± 0.10	0.19 ± 0.28	0.24
Snellen visual acuity, range	20/100–20/12	20/100–20/16	n.a
Intraocular pressure, mmHg	14.7 ± 2.7	14.5 ± 2.5	0.76
Serous retinal fluid height at the fovea, µm	140 ± 78	151 ± 101	0.70
Subfoveal choroidal thickness, µm	372 ± 100	352 ± 101	0.25
Reduced-fluence photodynamic therapy spot size, µm (range)	n.a	5390 ± 1159 (1851–6197)	

Data are shown as the mean ± standard deviation unless otherwise indicated.

*n.a.* not applicable, *rf-PDT* reduced-fluence photodynamic therapy, *logMAR* logarithm of the minimum angle of resolution.

Rf-PDT was performed for all affected eyes in the rf-PDT group. The mean diameter of the spot size for rf-PDT before treatment was 5390 ± 1159 µm. No systemic or ocular adverse events were observed following rf-PDT. After rf-PDT, the SRF height and subfoveal choroidal thickness in the rf-PDT group reduced to 33 ± 60 µm and 239 ± 56 µm, respectively. In contrast, in the control group, the SRF height decreased to 80 ± 65 µm; however, the subfoveal choroidal thickness remained unchanged at 370 ± 87 µm after an observational period of 1.2 ± 0.7 months. The difference in the period was due to the fact that the natural history of CSC was monitored at 1- to 2-month intervals, whereas follow-up after treatment was conducted within 1 month. Both the SRF height and subfoveal choroidal thickness in the rf-PDT group were significantly decreased compared with those in the control group at follow-up (*p* = 0.01 for foveal SRF height, *p* < 0.001 for subfoveal choroidal thickness; Table 2).

**Table 2:** Longitudinal changes in morphologic and functional parameters in the control group and the reduced-fluence photodynamic therapy group.

	Control group	rf-PDT group	*P* value
Observational period, months	1.2 ± 0.7	0.8 ± 0.5	< 0.001
Visual acuity in logMAR	0.05 ± 0.21	0.21 ± 0.28	0.04
Snellen visual acuity (range)	20/100–20/12	20/100–20/12	n.a
Serous retinal fluid height at the fovea, µm	80 ± 65	33 ± 60	0.01
Subfoveal choroidal thickness, µm	370 ± 87	239 ± 56	< 0.001

Data are shown as the mean ± standard deviation unless otherwise indicated.

*n.a.* not applicable, *rf-PDT* reduced-fluence photodynamic therapy, *logMAR* logarithm of the minimum angle of resolution.

Table 3 summarizes the comparisons of choroidal thickness in the nine subfields at baseline and at the follow-up time point in both groups. Relative to that before rf-PDT, the choroidal thickness after rf-PDT showed a significant decrease in not only all inner subfields but also all outer subfields at the posterior pole, including the vicinity of the vortex vein ampulla (*p* < 0.001 for all inner subfields; *p* = 0.035 and *p* = 0.024 for the outer superonasal and inferonasal subfields, respectively; *p* < 0.001 and *p* = 0.004 for the outer superotemporal and inferotemporal subfields, respectively). Contrarily, no significant difference was observed in the choroidal thickness across any subfields in the control eyes between the initial visit and the follow-up point.

**Table 3:** Comparisons of swept-source optical coherence tomography-examined wide-field choroidal thicknesses between baseline and follow-up in the control group and the reduced-fluence photodynamic therapy group.

Choroidal thickness	Control group			rf-PDT group		
	Baseline	Follow-up	*P* value	Baseline	After rf-PDT	*P* value
Central circle of 3-mm-diameter, µm	359 ± 80	362 ± 93	0.796	377 ± 88	322 ± 119	< 0.001
Grids enclosed by circles of 3-mm- and 9-mm-diameters						
Superonasal, µm	313 ± 77	313 ± 84	0.889	311 ± 89	293 ± 102	< 0.001
Inferonasal, µm	280 ± 90	275 ± 88	0.320	270 ± 80	248 ± 88	< 0.001
Superotemporal, µm	340 ± 66	334 ± 67	0.054	342 ± 76	304 ± 93	< 0.001
Inferotemporal, µm	332 ± 93	328 ± 100	0.465	317 ± 95	277 ± 102	< 0.001
Grids enclosed by circles of 9-mm- and 18-mm-diameters						
Superonasal, µm	278 ± 49	274 ± 48	0.080	232 ± 82	227 ± 87	0.035
Inferonasal, µm	248 ± 60	247 ± 62	0.594	167 ± 45	162 ± 46	0.024
Superotemporal, µm	257 ± 75	254 ± 74	0.158	270 ± 59	255 ± 64	< 0.001
Inferotemporal, µm	188 ± 56	187 ± 56	0.559	224 ± 60	209 ± 56	0.004

Data are shown as the mean ± standard deviation unless otherwise indicated.

*rf-PDT* reduced-fluence photodynamic therapy.

Our analysis revealed a significant positive correlation between the rate of reduction in choroidal thickness in the irradiated area and that in the non-irradiated area (correlation coefficient, 0.765; *p* < 0.001, Supplemental Figure).

## Discussion/Conclusion

Accumulating evidence has revealed that both choroidal thickening^6,7,16^ and choroidal vascular hyperpermeability^4^ in the macular area are essential clinical features of CSC. Recent studies using WF imaging demonstrated additional choroidal features of CSC, such as vertically and asymmetrically dilated choroidal veins including the vortex vein ampulla^10^, choroidal anastomosis at the watershed^11^, and choroidal pulsations^28^.

Initial evidence from Chan et al.^21^ suggested the efficacy of hd-PDT for acute CSC. Thereafter, various investigators reported the efficacy of hd/rf-PDT for chronic or recurrent CSC^23,29^. Using OCT or ICGA, several studies have shown post-PDT changes in the choroidal structures in eyes with CSC^21,24,25,30^. Using ICGA, Chan et al.^21,24^ showed a reduction in the diameter of the macular choroidal vessels as well as the choroidal vascular leakage in CSC following PDT. Maruko et al.^31^ showed that subfoveal choroidal thickness and choroidal vascular hyperpermeability were reduced in CSC after hd-PDT. Izumi et al.^25^ showed a marked reduction in the thickness of the large choroidal vessel layer, but not of the choriocapillaris-medium choroidal vessel layer, after hd-PDT. However, previous studies focused on the examination of the choroidal changes within the macular area where the PDT laser was irradiated. This study compared the choroidal thickness at the posterior pole before and after rf-PDT in eyes with CSC using EDI of WF SS-OCT. Previous research by Sugano et al.^32^ reported that changes in the subfoveal choroidal thickness mirrored those in the choroidal thickness outside the laser-irradiated area after PDT in eyes with polypoidal choroidal vasculopathy. Consistent with the findings of that study, our study found a positive correlation between choroidal thickness changes in the irradiated area and those in the non-irradiated areas (Supplemental Figure). However, our study was different in that it focused on eyes with CSC, and two-dimensional evaluation of the region up to the vicinity of the vortex vein was possible.

Histopathologic evidence demonstrates that PDT selectively occludes the choriocapillaris in targeted areas^33^. With its lower irradiation energy, rf-PDT is presumed to induce even fewer morphological and functional changes within the choriocapillaris^34^. This leads us to propose that the effectiveness of rf-PDT arises from a moderated reduction in choriocapillaris blood flow in the irradiated region, resulting in a similar moderated decrease in the blood flow of the short posterior ciliary artery, given its constant pressure and increased resistance.

Recent studies utilizing WF choroidal thickness maps have shown choroidal thickening from the vortex vein ampulla along the dilated vortex veins in eyes with CSC^12^. This suggests that the pathogenesis of CSC may be associated with impaired drainage of the vortex veins at the sclera. In CSC, it is believed that overload or congestion in the choroid leads to increased permeability at the choriocapillaris, which results in the formation of SRF at the posterior pole^10,16^. In the present study, we observed that rf-PDT resulted in a substantial reduction in choroidal thickening, not only in the irradiated macular area but also outside the vascular arcade (Table 3, Figs. 2, 3).

Considering the substantial blood flow in the macular choriocapillaris^9,35^ and its upstream position in the choroidal circulation^36^, it is plausible that it could influence the outflow into the vortex veins across all quadrants. Accordingly, we suggest that rf-PDT reduces SRF in CSC by selectively curtailing blood flow in the choriocapillaris within the irradiated area^33,34^ and by decreasing the volume of blood entering the vortex veins. This action could alleviate vortex vein congestion and reduce SRF at the posterior pole.

This study has several limitations. First, the number of patients included in the study was limited. Second, the follow-up period was short although the duration was comparable to a previous report^31^. Third, this study did not determine the precise cause of the observed decrease in the peripheral choroidal thickness. This reduction could be attributed to suppression of the increased permeability of choroidal vessels by PDT. Nevertheless, considering that the reduction in the choroidal thickness was much more extensive than the PDT-applied area, we speculate that this effect might primarily be due to the alleviation of overload at the vortex venous ampulla. Future research is planned to prospectively investigate the actual factors contributing to this extensive reduction in choroidal thickness after rf-PDT. Fourth, because this was a retrospective study, the observational period in the control group was longer than that in the rf-PDT group. Finally, WF SS-OCT images may have been overestimated at the periphery because the measurements were made obliquely to the choroidal interface at the periphery. However, as we compared the same subfields of the same eye before and after treatment, possible distortions in the measurement of choroidal thickness at the periphery did not seem to be significant enough to overturn our conclusions.

In conclusion, eyes with CSC showed WF choroidal thickening from the ampulla of dilated vortex vein to the fovea along the vascular course. However, rf-PDT achieved an extensive reduction in the choroidal thickness not only in the irradiated macular area but also outside the arcade vessels in all quadrants. These findings provide new insights into the pathogenesis of CSC and explain the reasons for the effectiveness of rf-PDT for this condition. This can pave way for the development of best-practice guidelines for the treatment of CSC.

## Methods

### Patients and methods

This observational study received approval from the Institutional Review Board of Kyoto University Graduate School of Medicine, Kyoto, Japan and adhered to the principles of the Declaration of Helsinki. Written informed consent was obtained from all participants before the study procedures and examinations were initiated.

Patients with treatment-naïve unilateral CSC were enrolled at Kyoto University Hospital between December 2021 and July 2022 (ClinicalTrials.gov identifier, NCT02081339). The diagnosis of CSC was based on the presence of SRF at the posterior pole, hyperfluorescent leakage areas on fluorescein angiography (FA) with RPE damage, and choroidal vascular hyperpermeability appearing as hyperfluorescent in the middle-phase ICGA. Exclusion criteria included previous treatment for CSC, corticosteroid use, and intraocular surgery other than cataract surgery as described previously^28^. Patients with other choroidal neovascularization based on OCT and FA/ICGA results that may affect foveal structures, as well as those with poor-quality OCT images were also excluded. Finally, 20 eyes of 20 consecutive patients were included in the study. In addition, we retrospectively analyzed the chart records of 20 consecutive patients adjusted for age and sex as a control group. The same analytical process applied to the eyes with CSC treated with rf-PDT was employed for the control group.

Each patient underwent a comprehensive ophthalmic examination, including refraction measurement, decimal best-corrected visual acuity (BCVA) measurement, measurement of intraocular pressure and axial length, slit-lamp biomicroscopy, color fundus photography, FA, ICGA, fundus autofluorescence photography, and SS-OCT. Using the Early Treatment Diabetic Retinopathy Study (ETDRS) grid, we defined the subfoveal choroidal thickness as the averaged choroidal thickness in the central grid (1 mm in diameter). Objective refraction was measured with an autorefractor, and the axial length was measured with an interferometer (IOL Master 700; Carl Zeiss Meditec, Dublin, CA, USA). The spherical equivalent was calculated as the sum of the spherical power and half of the cylindrical power. Color fundus photography was performed using a fundus camera system. FA and ICGA were performed using a confocal scanning laser ophthalmoscope as previously described^12^.

### reduced-fluence photodynamic therapy

In this study, all 20 patients in the rf-PDT group underwent treatment with a standard dose of verteporfin (6 mg/m^2^) that was intravenously administered over a span of 10 min. Laser treatment was performed exactly 15 min after the drug infusion, with a reduced fluence of 25 J/cm^2^ and an exposure duration of 83 s. The laser was delivered to cover both, areas with the leak point based on FA and the choroidal vascular hyperpermeability on ICGA^29^ that had been observed before treatment. In this study, all PDT spots were within a circle with an approximate diameter of 7 mm on the temporal side of the disc. After treatment, patients were instructed to avoid exposure to sunlight for 48 h. rf-PDT was recommended for CSC diagnosed by multimodal imaging, with reference to the PLACE trial^37^. Specifically, rf-PDT was used for eyes with SRF that was observed to affect the fovea on OCT, as well as those with focal or diffuse hyperfluorescent leakage areas, an RPE window defect observed on FA, hyperfluorescent changes on ICGA, and RPE abnormality on fundus autofluorescence.

In this study, patients with CSC who met the criteria for the PLACE study were indicated for rf-PDT. However, in clinical settings, some patients are unable to undergo treatment or refuse treatment for various reasons. Therefore, for each individual case, the final decision was made based on discussions with macular specialists after considering individual patient factors such as BCVA, symptom duration, number of recurrences, the ophthalmic status determined by multimodal imaging data, and consent for treatment.

### Evaluations of choroidal thickness by EDI of WF SS-OCT

We examined the choroidal structure using SS-OCT (Xephilio OCT-S1, Canon Medical Systems, Otawara, Japan) with 1010–1110-nm near-infrared light (scanning laser ophthalmoscope, 780 nm) and a scanning speed of 100,000 A-scans/s. No supplementary equipment or lenses were required for image acquisition.

To measure changes in choroidal thickness on WF, the scanning area was captured at 20 mm (vertical, 128 B-scans) and 23 mm (horizontal, 1024 pixels) with a 5.3-mm scan depth (1396 pixels) for 3D volumetric data using EDI in SS-OCT. The choroidal thickness was defined as the vertical distance from the Bruch’s membrane to the choroidoscleral border which was evaluated by the built-in software supported by artificial intelligence. When automatic machine segmentation of the inner and outer edges of the choroid was performed inappropriately, the segmentation errors were corrected manually as needed.

In this study, the choroidal thickness was measured using a grid consisting of three circles of 3, 9, and 18 mm diameter, respectively (Fig. 1)^12^. The inner and outer rings were defined as the fields enclosed between the 3- and 9-mm circles, and between the 9- and 18-mm circles, respectively. Each ring was divided into four subfields, superotemporal, inferotemporal, superonasal, and inferonasal quadrants, respectively. Measurements were performed over a total of nine subfields together with the central 3-mm ring (Fig. 1). We measured the choroidal thickness in each subfield both before and after rf-PDT during the first visit to the outpatient clinic. For the control group, measurements were taken during the initial visit and the subsequent follow-up visit without treatment.

Where choroidal thickness was measured, we corrected for axial length-related magnification using the measurements of axial length in the modified Littmann formula (Bennett procedure)^12,38^.

In the rf-PDT group, we explored the correlation between the reduction in choroidal thickness in areas irradiated with rf-PDT and that in non-irradiated areas.

### Statistical analysis

Statistical analyses were performed using JMP 16 software (SAS Institute Inc., Cary, NC, USA). All values were presented as mean ± standard deviation. The difference in sex distribution between the rf-PDT group and the control group was evaluated using the chi-square test. Other comparisons between the two groups were performed using the Mann–Whitney U test. The mean foveal thickness, SRF height at the fovea, and subfoveal choroidal thickness for each sector were compared between different observational points using the Wilcoxon signed-rank test. The correlation between the reduction in choroidal thickness in areas irradiated with rf-PDT and the reduction in choroidal thickness in non-irradiated areas was evaluated by the Spearman rank test. To quantify the visual changes, a Landolt chart was converted to the logarithm of the minimum angle of resolution (logMAR) BCVA for analysis. Statistical significance was set at *p* < 0.05.

## Supplementary Information

Supplementary Figures.

Supplementary Legends.

## Data Availability

The datasets generated during and/or analyzed during the current study are available from the first author [Naomi Nishigori] on reasonable request.
