# Supplementary material for: Extensive reduction in choroidal thickness after photodynamic therapy in eyes with central serous chorioretinopathy
Source: Sci Rep. 2023 Jul 5;13:10890. doi: 10.1038/s41598-023-37802-w (PMC10322984; doi:10.1038/s41598-023-37802-w)
Supplement: Supplementary file 2 — Supplementary Legends. [file 41598_2023_37802_MOESM2_ESM.docx]

**Supplemental Figure. 1.** **Association between the reduction in choroidal thickness in areas irradiated with reduced-fluence photodynamic therapy and that in areas outside those irradiated with reduced-fluence photodynamic therapy**

The rate of choroidal thickness reduction in the irradiated area shows a significant positive correlation with that in the non-irradiated area in the reduced-fluence photodynamic therapy group. The correlation coefficient was 0.765 (p < 0.001).
